# Supplementary material for: lncRNA MSTRG4710 Promotes the Proliferation and Differentiation of Preadipocytes through miR-29b-3p/IGF1 Axis
Source: Int J Mol Sci. 2023 Oct 28;24(21):15715. doi: 10.3390/ijms242115715 (PMC10649235; doi:10.3390/ijms242115715)
Supplement: Supplementary file 1 [file ijms-24-15715-s001.zip › ijms-2652789-supplementary.pdf]

1	TCCACAAAACATTCTCCTTTCCTTCTGAAGGTTTTACGATGCATTATCATTAACCAGTCTTTGAT	65
66	TGGGTCGTTTCGCTGTCCGTGATAGGAGACAACCAGTTCTGAGACCGTTCTTCCCCCACTGATTA	130
131	AGACTGGGGTGGCAGGTATTAGGGGTAATATTCATTTAGCCTTCTGAGCTTTCTGGGCAGACTTG	195
196	GTGACCTTGCCAGCTCCAGCAGCCTTCTTGTCCACTGCTTTGATCACACCCACAGCAACCGTCTG	260
261	TCTCATATCGCGGACAGCGAAACGACCCAATCTCTAGAGGATCCCCGGGTACCGAGCTCGAATTC	325
326	ACTGGCCGGTCGGTCACATATCAACAATGGCAGCATCACCAGATTTCAAGACTTCAGCTCAGCAA	390
391	ACTTGCAAGCAATGTGAGCTGTGTGACAATCCAGTACAGGGGCATACCCAGCACTGATCTGACCT	455
456	GGATGGTTCAGGATGATCACCTGAGCAGTGAAGCCAGCTGCTTCCATTGGTGGGTCATTTTTGCT	520
521	GTCACCAGCAATGTTGCCACGAGGAACATCTTTGACAGACACGTTCTTGACATTGAAGCCCACAT	585
586	TGTCCCCAGGAAGAGCTTCACTCAAAGCTTCATGGTGCATTTCGACGGACTTGACTTCAGTTGTG	650
651	ACATTGACTGGAGCAAAAGTTACCACCATGCCAGGTTTGAGAACACCAGTCTCCACTCGGCCCAC	715
716	AGGGACAGTGCCAATACCACCAATTTTGTAGACATCCTGTAGGGGCAGACGCAGAGGCTTGTCAG	780
781	TTGGTCTAGTTGGTGGAAGGATGCAGTCCAGGGCTTCAAGCAGTGTGGTTCCACTGGCATTGCCA	845
846	TCTTTGCGGGTGACTTTCCATCCCTCAAACCATGGCATATTAGCACTTGGCTCCAGCATGTTGTC	910
911	ACCGTTCCAACCAGAAATTGGCACAAATGCTACTGCGTCAGGGTTGTAGCCAATTTTCTTAATGT	975
976	AGGTGCTGACTTCCTTAACGATTTCCTCGTATCTCTTCTGGCTGTAGGGTGGCTCAGTGGAATCC	1040
1041	ATCTTGTTAACACCAACCATTAGCTGTTTCACACCCAGCGTGTAAGCCAGAAGGGCATGCTCACG	1105
1106	GGTCTGCCCGTTCTTGGAGATACCAGCTTCAAATTCCCCGACACCAGCAGCAACAATCAAGACGG	1170
1171	CACAGTCAGCCTGAGATGTGCCTGTAATCATGTTTTTGATGAAGTCTCTGTGTCCTGGGGCATCA	1235
1236	ATGATAGTCACGTAGTACTTGCTGGTCTCAAATTTCCACAGGGAGATGTCGATGGTGATACCACG	1300
1301	CTCACGCTCGGCTTTCAGTTTATCCAAGACCCAGGCACACTTGAAGGAGCCCTTTCCCATCTCGG	1365
1366	CAGCCTCCTTCTCAAATTTTTCAATGGTTCTTTTGTCGATGCCACCACATTTGTAGATCAGATGG	1430
1431	CCAGTGGTGGTGGACTTGCCCGAATCTACGTGGCCAATGACGACGATGTTGATGTGAGTCTTTTC	1495
1496	CTTTCCCATTTTGGCTTTGATTTAGCGGTGGTTTTCACGACACCTGTGTTCTGGCGGCAAACCCG	1560
1561	TTACGTAGCGTATCGTTGACAGC	1583

Figure S1. Full sequence of MSTRG4710.

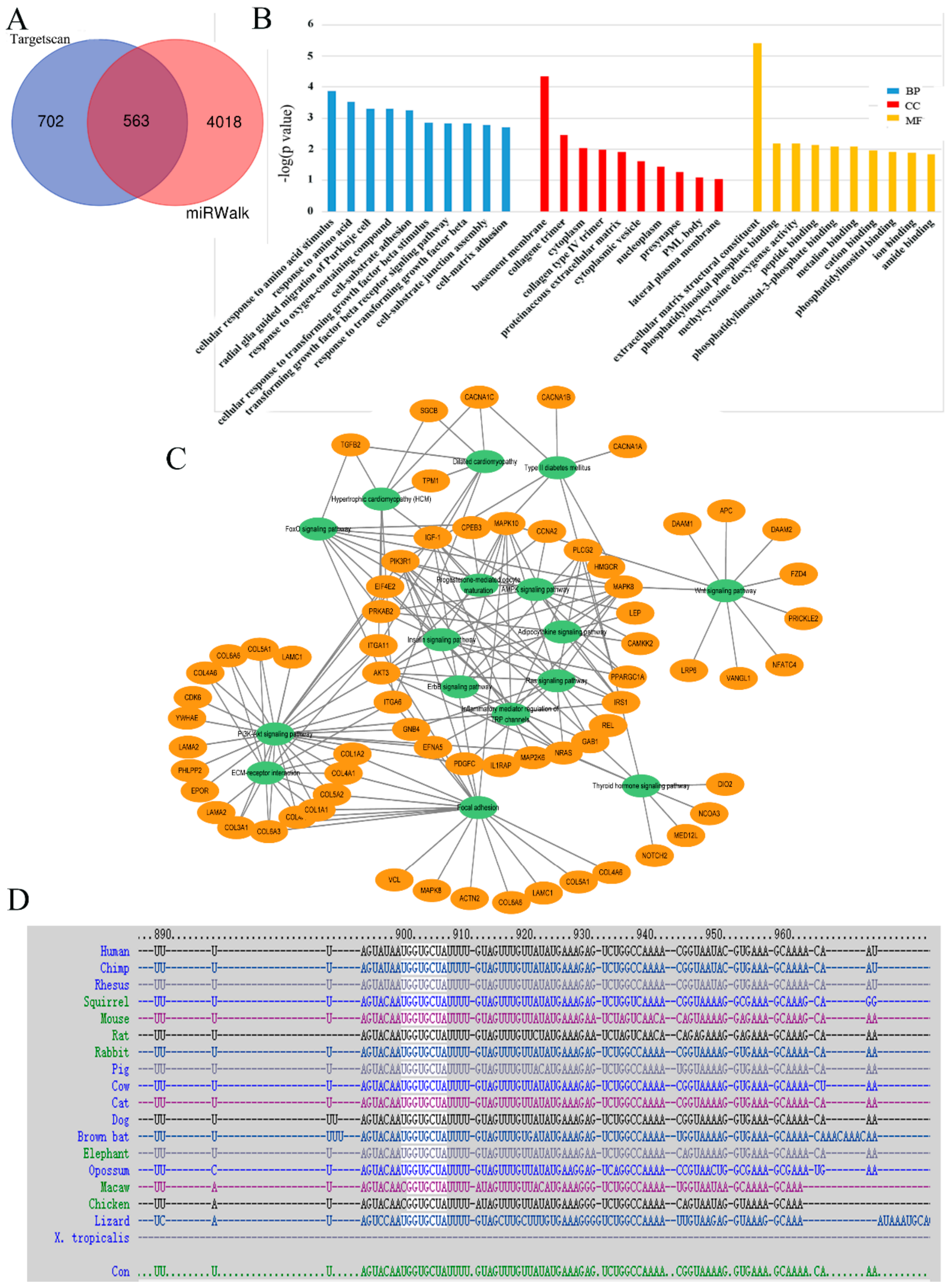

Figure S2. Prediction of miR-29b-3p target gene. (A) Venn diagram of target gene prediction results; (B) GO enrichment analysis of target genes. BP means biological process. CC means cellular component. MF means molecular function. (C) Network diagram of relationship between target gene and pathway. The green ellipse represents the KEGG path. The orange ellipse represents the gene. (D) Prediction of target gene binding sites. Binding sites are indicated by white areas.

Table S1. The primer sequences used for MSTRG4710 RACE

**Name**	**Primer**
MSTRG4710	TAATGTAGGTGCTGACTTCC
MSTRG4710-5'-F1	CAGGCTGACTGTGCCGTCTTGATTG
MSTRG4710-5'-F2	CTCCTCTGGGTCGTTTCGCTGTCCG
MSTRG4710-3'-F1	TTGGCACAAATGCTACTGCGTCA
MSTRG4710-3'-F2	CAGCAGCAACAATCAAGACGGCAC
UPM	CTAATACGACTCACTATAGGGCAAGCAGTGGTATCAACGCAGAGT
3'Midp	GCTGTCAACGATACGCTACGTAACG
3'Insp	CGCTACGTAACGGCATGACAGTG

Table S2. The primer sequences used for the qRT-PCR analysis.

**Name**	**Gene ID**	**Forward primer (5′-3′)**	**Reverse primer (5′-3′)**
miR-29b-3p		UAGCACCAUUUGAAAUCAGUGU	
GAPDH	100349551	CTTCGGCATTGTGGAGGG	GGAGGCAGGGATGATGTTCT
C/EBPα	100358916	CAAGAACAGCAACGAGTACCG	GTCACTGGTCAACTCCAGCAC,
PPARγ	100008892	GAGGACATCCAGGACAACC	GTCCGTCTCCGTCTTCTTT
FABP4	100009416	CTAGATGGTGGTGCCCTGGT	AGTTTATCGCCCTCCCGTTT
SREBP1	100008784	CACTTCATCAAGGCGGACTCG	TAGTCGGTGGATGGGCAGTTT
PCNA	100339381	TTGCACGTATATGCCGAGACC	GGTGAACAGGCTCATTCATCTCT
CDK2	100134865	TTTGCCGAGATGGTGACCC	CAGAGCTGCCTTTGCCGAAA
CDK3	100337942	GGTGTTTGAGTTCCTCAGCC	TTGACTAGGTGCATGGGCAG
CDK4	100009039	AGTTTCTAAGCGGCCTGGAT	AACTTCAGGAGCTCGGTACC
MSTRG4710		TAATGTAGGTGCTGACTTCC	TGACTGTGCCGTCTTGAT
U6		CTCGCTTCGGCAGCACA	AACGCTTCACGAATTTGCGT
